# Case Report: Use of Obinutuzumab as an Alternative Monoclonal Anti-CD20 Antibody in a Patient With Refractory Immune Thrombocytopenia Complicated by Rituximab-Induced Serum Sickness and Anti-Rituximab Antibodies

**DOI:** 10.3389/fimmu.2022.863177

**Published:** 2022-04-19

**Authors:** Jennifer R. Blase, David Frame, Thomas F. Michniacki, Kelly Walkovich

**Affiliations:** ^1^ Department of Pediatrics, Division of Hematology/Oncology, University of Michigan, Ann Arbor, MI, United States; ^2^ Department of Pharmacy, University of Michigan, Ann Arbor, MI, United States

**Keywords:** serum sickness, ITP (idiopathic thrombocytopenic purpura), rituximab, obinutuzumab, case report

## Abstract

Management of refractory immune thrombocytopenia frequently involves rituximab, a chimeric anti-CD20 monoclonal antibody, to target B cells and induce remission in most patients. However, neutralizing antibodies to rituximab that nullify therapeutic response and may lead to serum sickness have been rarely reported. Here, we present a case of a young adult woman with Evans syndrome treated with rituximab, complicated by the development of serum sickness, acute respiratory distress syndrome, and platelet refractoriness presumed secondary to neutralizing antibodies to rituximab. She was successfully treated with the humanized anti-CD20 monoclonal antibody, obinutuzumab, with subsequent symptom resolution. Additionally, a review of 10 previously published cases of serum-sickness associated with the use of rituximab for idiopathic thrombocytopenic purpura (ITP) is summarized. This case highlights that recognition of more subtle or rare symptoms of rituximab-induced serum sickness is important to facilitate rapid intervention.

## Introduction

Idiopathic thrombocytopenic purpura (ITP) arises from immune clearance or suppression of platelets. Corticosteroids and intravenous immunoglobulin (IVIG) are commonly used in the first-line management of newly diagnosed ITP. However, management of refractory or chronic ITP frequently relies on the use of anti-CD20 monoclonal antibody therapy, most commonly rituximab, a type 1 chimeric IgG antibody (1). Rituximab reversibly depletes CD20+ B cells and induces remission in 52%–73% of patients with ITP through the cessation of antibodies directed against platelet-surface glycoproteins (2). Relapse of ITP is common; however, retreatment is often successful, as 80% of patients respond to repeat rituximab courses (3).

In general, rituximab is well-tolerated apart from a common first-dose infusion reaction that is primarily due to rapid cytokine release because of brisk destruction of B-cell targets by the monoclonal antibody. Infusion reactions should not be confused with the rarer type III immune-complex-mediated hypersensitivity reaction that may occur from anti-rituximab antibodies and often results in rituximab-induced serum sickness (RISS). Prevalence of RISS is reported at high rates in patients with systemic autoimmune disorders, as high as 39% in patients with systemic lupus erythematosus (4). In children with ITP, the prevalence is lower, reported to be between 6% and 12% (5, 6). RISS may often be under-recognized, especially with earlier infusions, as less than half of patients present with the classic triad of fever, rash, and arthralgias (7).

Prompt recognition of RISS and initiation of corticosteroids are important in the management of ITP patients, particularly as re-exposure to rituximab is common and may trigger more severe clinical manifestations such as anaphylaxis (8). Newer humanized (e.g., obinutuzumab) and fully human (e.g., ofatumumab) monoclonal anti-CD20 antibodies exist that may have less risk of serum sickness without cross-reacting with rituximab but have rarely been employed in the treatment of ITP (9).

Here we report a 25-year-old patient treated with rituximab complicated by the development of serum sickness, acute respiratory distress syndrome (ARDS), and platelet refractoriness presumed secondary to neutralizing antibodies to rituximab successfully treated with obinutuzumab. Additionally, a review of 10 previously published cases of serum sickness associated with the use of rituximab for ITP is summarized.

## Case Description

**Figure 1:**
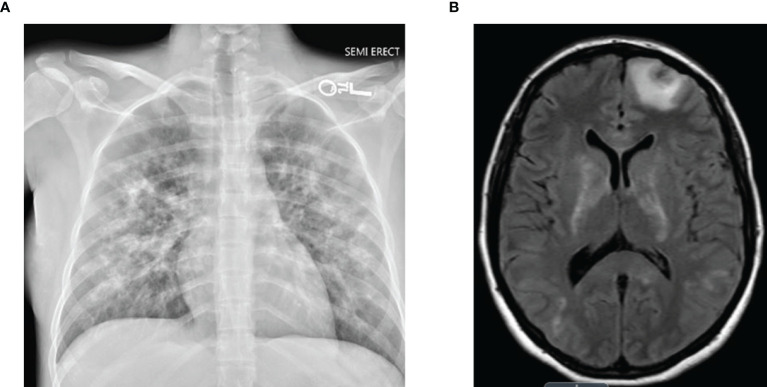
**(A)** Chest X-ray revealing diffuse interstitial and airspace opacities. **(B)** Brain MRI revealing extensive fluid-attenuated inversion recovery (FLAIR) signal abnormalities throughout supratentorial parenchyma.

A 25-year-old woman with relapsing–remitting Evans syndrome presented with refractory severe thrombocytopenia and grade III mucosal bleeding despite prednisone, intravenous IVIG (1 g/kg × 3 doses), romiplostim (10 μg/kg), and rituximab. Her CD20+ B-cell counts remained normal despite 100 mg/m^2^ × 3 doses and 375 mg/m^2^ × 2 doses of rituximab. Eighteen days after her first rituximab dose, she reported new-onset severe neuropathic pain in her right leg diagnosed as piriformis syndrome. Subsequently, she developed fevers, malaise, arthralgias, blurry vision, and abrupt acute hypoxic respiratory failure with intracranial hemorrhages requiring mechanical ventilation (**Figure 1**). While her thrombocytopenia was associated with petechiae, no other discrete rash was observed. Her arthralgias began 5 days after her third rituximab dose, fevers started 17 days after her fifth rituximab dose, and respiratory symptoms developed 18 days after her fifth rituximab dose. Extensive evaluation for infectious etiologies of her fever and ARDS was negative. Malignancy screening, including a bone marrow biopsy, was negative for lymphoproliferative disorders. Additionally, further evaluation with whole-exome sequencing for underlying inborn errors of immunity and screening for systemic autoimmune disorders was non-diagnostic. Of note, she was previously treated with rituximab 375 mg/m^2^ × 4 doses four years prior for ITP without incident. However, repeat dosing for an ITP relapse one year prior with rituximab 100 mg/m^2^ × 4 doses was complicated by an infusion reaction with her initial dose (bronchospasm requiring treatment with hydrocortisone, famotidine, and albuterol). She also reported fatigue and jitteriness following her third and fourth doses that improved with corticosteroids with early B-cell recovery within 2 months.

**Figure 2:**
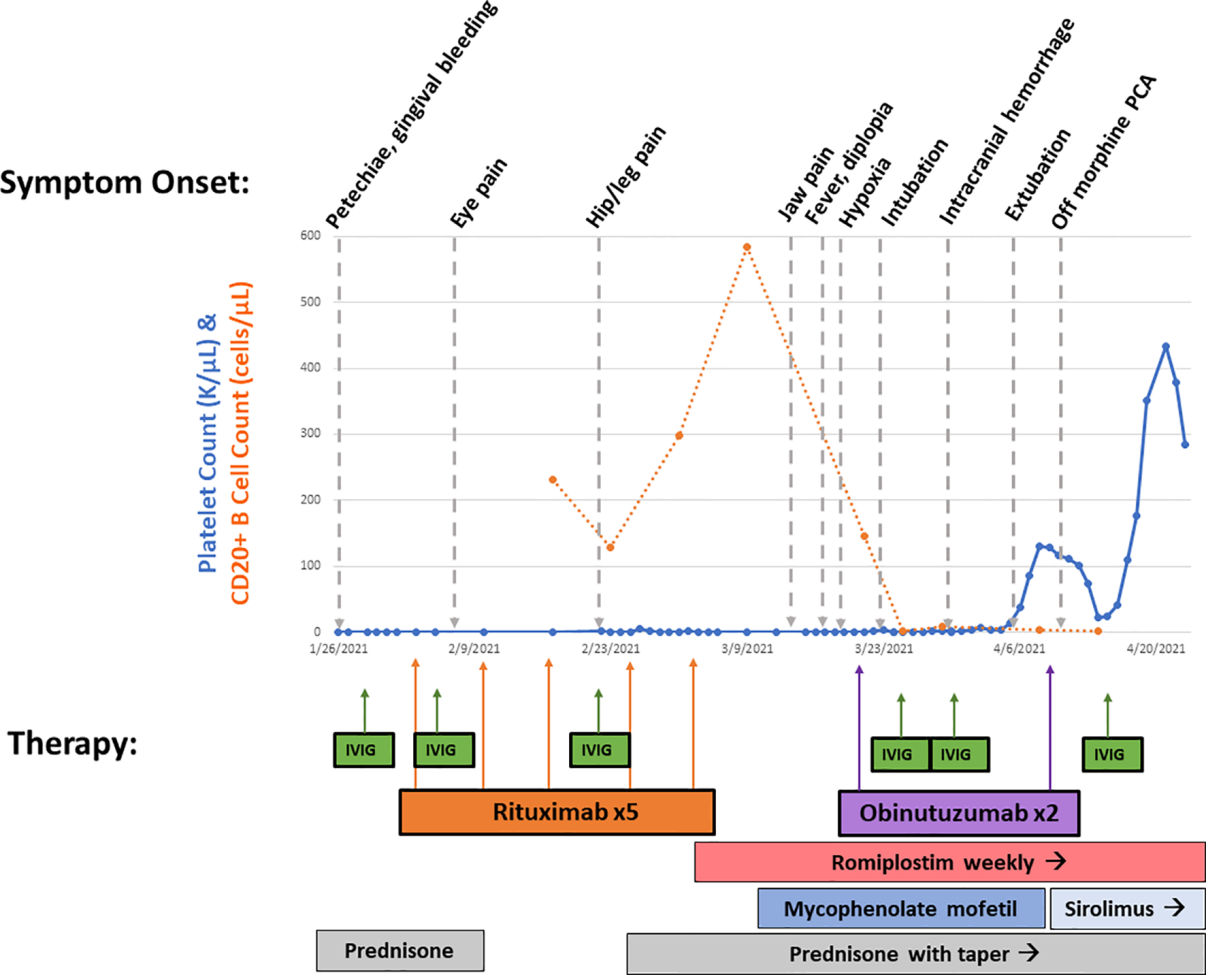
Overview of treatment course. Platelet count (in K/µl, solid line) and CD20+ B-cell count (in cells/µl, dotted line) from beginning of presentation until hospital discharge is shown. Symptom onset is shown on top, while therapies are shown on bottom with corresponding arrows. This demonstrates rapid B-cell depletion following obinutuzumab therapy with gradual rise in platelet count.

Suspecting neutralizing anti-rituximab antibodies, which were later confirmed [LapCorp rituximab drug level <0.2 µg/ml, anti-rituximab antibodies 4,502 mg/ml (normal < 25)], she received two doses of obinutuzumab 1,000 mg/m^2^ with rapid depletion of her CD20+ B cells. Given the severity of her bleeding, she was concurrently retreated with IVIG, continued on high-dose corticosteroids and romiplostim, and initiated on mycophenolate mofetil that was subsequently transitioned to sirolimus (**Figure 2**). She had gradual resolution of her thrombocytopenia. Her ARDS improved following administration of obinutuzumab, and her severe neuropathic pain, despite being previously refractory to multitherapy treatment with gabapentin, cyclobenzaprine, and opioids, improved following obinutuzumab administration coincident with her platelet count stabilizing. Since multiple therapeutic interventions were initiated concurrently, we are unable to definitely determine which agent led to improvement. There was a decrease in B-cell count following mycophenolate mofetil treatment; however, the abrupt and complete elimination of B cells did not occur until after obinutuzumab administration. She remains on sirolimus with intermittent doses of romiplostim to maintain a platelet count >50,000 cells/µl and has documented the repopulation of her CD20+ B cells.

## Discussion

While more commonly reported in systemic autoimmune disease, RISS remains a rarely reported complication of therapy in ITP despite the broad use of rituximab as therapy for chronic or refractory ITP (**Table 1**). Details for some of the previously published cases are sparse, but cases of infants through adults (8 months to 48 years) are reported. As shown (**Table 1**), 7/8 (87.5%) ITP patients with available symptom data were documented to have the classic triad symptoms of fever, rash, and arthralgias. Malaise and fatigue were also reported in 5/8 (62.5%) ITP patients with available symptom data.

**Table 1:** Previously published case reports of rituximab-induced serum sickness in patients with immune thrombocytopenia.

Publication	No. pts.	Age/gender	No. doses	Concurrent diagnoses	Presence of anti-rituximab antibodies?	Classic symptoms			Other features	RISS treatment?
						Fever?	Rash?	Arthralgias?		
Godeau et al. (10)	1	NA^§^	2	NA	NA	NA^§^	NA	NA	No	None
Wang et al. (6)	3	14F	2	NA	NA	Yes	Yes	Yes	Malaise	NA
		12F	3	NA	NA	No	No	Yes	Rash during dose 1	NA
		12F	1	NA	NA	Yes	Yes	Yes	Malaise	NA
Bennett et al. (5)	2	12M	2	NA	NA	Yes	Yes	No	Fatigue	NA
		11F	2	NA	NA	Yes	Yes	Yes	Conjunctival hyperemia	NA
Goto et al. (11)	1	8M	2	No	Yes	Yes	Yes	Yes	Fatigue and rash developed 11 days after fever, arthralgias	Prednisolone
Medeot et al. (12)	1	NA^¶^	2	NA	NA	NA^¶^	NA	NA	No	None
Herishanu et al. (13)	1	48F	2	No	NA	Yes	Yes	Yes	Malaise	Methyl-prednisolone
Manko et al. (14)	1	46F	1	Asthma	NA	Yes	Yes	Yes	Hypoxemic respiratory failure (ARDS)	IVIG + plasmapheresis

NA, not available; RISS, rituximab-induced serum sickness; ARDS, acute respiratory distress syndrome; IVIG, intravenous immunoglobulin.

^§^Information not available, but in an adult cohort (18–84) and listed symptoms as transient serum sickness.

^¶^Information not available, but in an adult cohort (18–76) and listed as grade 3 serum sickness with rapid improvement.

Clinically, it is important to differentiate between the more commonly observed rituximab-related infusion reaction and serum sickness, as each occurs due to a unique immune mechanism, therefore requiring different management approaches. Infusion reactions are primarily noted with the first infusion and are more commonly reported in patients with hematologic malignancies as compared to those with autoimmune conditions (15). Patients often report fever, chills, rigors, pruritus, nausea, headache, or less commonly hypotension, hypoxia, and bronchospasm. Typically, infusion-related reactions are secondary to cytokine release (15) and can be managed with acetaminophen, antihistamine, and corticosteroids as needed or as pre-medications. RISS, however, generally develops 1–2 weeks after exposure to the offending agent and may be within a few days of subsequent doses. The long half-life of rituximab also increases the risk of late symptoms occurring. Serum sickness occurs when excess non-human or heterologous antigens, such as murine Fab fragments of rituximab, bind to circulating IgG antidrug antibodies and form immune complexes. This occurs more commonly with chimeric antibodies, which contain more foreign antigens than humanized or human antibodies (16). Generally, the intermediate-sized immune complexes that deposit in vessel walls and tissues result in the activation of complement, granulocytes, and macrophages that trigger inflammation, increased vascular permeability, and tissue damage. The most frequently observed symptoms include fever, rash, and arthralgias, although less common symptoms include headache/blurry vision, edema, lymphadenopathy, splenomegaly, peripheral neuropathy, nephropathy, and/or vasculitis (17). Hypocomplementemia is also frequently observed in serum sickness (17), and in fact, the C4 complement level was low in our patient, but C3 was normal (C4 10 mg/dl, C3 147 mg/dl). Additionally, the development of ARDS in the case reported by Manko et al. (14), as well as in our patient, is postulated to be a consequence of immune-complex deposition leading to alveolar damage and vascular leak. While mild cases can be managed with non-steroidal anti-inflammatory drugs and/or antihistamines, severe cases require corticosteroids and consideration of IVIG and/or plasmapheresis (14).

When RISS occurs, it prompts the decision to stop therapy; however, it may be possible to change to the humanized type II anti-CD20 antibody, obinutuzumab, as used in our patient, or to the human type I antibody, ofatumumab (18). Since rituximab and ofatumumab are both type I antibodies, they rely more on complement-dependent cytotoxicity (CDC) with ofatumumab notably engineered to be even more dependent on CDC (19). Complement is often depleted from consumption with type III hypersensitivity reactions. Thus, we chose obinutuzumab for treatment, as it is a type II antibody that is glycoengineered to work primarily through antibody-dependent cellular cytotoxicity (ADCC) and direct cytotoxic mechanisms. Theoretically, there could be a slightly higher potential of cross-reactivity with rituximab and the humanized antibody. However, one small report did not see a difference between the drugs (19). Unfortunately, even fully human antibodies, which have no mouse component, may be immunogenic, especially in patients with autoimmune disorders that may have a defect in tolerance mechanisms (20).

Given the rarity of RISS in ITP patients, it is critical to maintain a high index of suspicion, particularly in higher-risk patients such as those receiving multiple courses of rituximab and those with an underlying systemic autoimmune disease. Perhaps even more problematic is the presence of neutralizing anti-rituximab antibodies, which may not elicit an immune response and result in a faster B-cell reconstitution or, as in this case, no clearance of B cells. Neutralizing antibodies may contribute to the 20%–40% non-response or loss of response to rituximab in many disorders. This poses the question as to whether an assessment of rituximab activity should be routinely monitored. While possible to measure anti-rituximab antibodies, it generally requires samples to be sent out, and the turnaround time may limit practical utility. Flow cytometry to measure B-cell levels is more accessible but less specific. While rituximab has become a routine agent in patients for non-oncologic purposes, it is critical that recognition of more subtle or rare symptoms of RISS is appreciated to facilitate rapid intervention, most importantly drug discontinuation, and to either try desensitization protocols or prompt transition to alternate therapy for the underlying disorder (21).

## Data Availability Statement

The original contributions presented in the study are included in the article/supplementary material. Further inquiries can be directed to the corresponding author.

## Ethics Statement

Written informed consent was obtained from the individual(s) for the publication of any potentially identifiable images or data included in this article.

## Author Contributions

JB and KW: conception and design. JB, DF, TM, and KW: writing, review, and revision of the manuscript. All authors read and reviewed the manuscript and contributed to and approved the final version as submitted.

## Conflict of Interest

The authors declare that the research was conducted in the absence of any commercial or financial relationships that could be construed as a potential conflict of interest.

## Publisher’s Note

All claims expressed in this article are solely those of the authors and do not necessarily represent those of their affiliated organizations, or those of the publisher, the editors and the reviewers. Any product that may be evaluated in this article, or claim that may be made by its manufacturer, is not guaranteed or endorsed by the publisher.
